# Intent among Parents to Vaccinate Children before Pediatric COVID-19 Vaccine Recommendations, Minnesota and Los Angeles County, California—May–September 2021

**DOI:** 10.3390/vaccines10091441

**Published:** 2022-09-01

**Authors:** Kara A. Suvada, Stuart F. Quan, Matthew D. Weaver, Meera Sreedhara, Mark É. Czeisler, Kathy Como-Sabetti, Ruth Lynfield, Prabhu Grounder, Elizabeth Traub, Aryana Amoon, Chandresh N. Ladva, Mark E. Howard, Charles A. Czeisler, Shantha M. W. Rajaratnam, Donatus U. Ekwueme, Brendan Flannery, Rashon I. Lane

**Affiliations:** 1 Department of Epidemiology, Rollins School of Public Health, Emory University, Atlanta, GA 30322, USA; 2 CDC COVID-19 Response Team, Centers for Disease Control and Prevention, Atlanta, GA 30329, USA; 3 Division of Sleep and Circadian Disorders, Brigham and Women’s Hospital, Harvard Medical School, Boston, MA 02115, USA; 4 Division for Heart Disease and Stroke Prevention, Centers for Disease Control and Prevention, Atlanta, GA 30329, USA; 5 Institute for Breathing and Sleep, Austin Health, Heidelberg 3010, Australia; 6 Turner Institute for Brain and Mental Health, Monash University, Clayton 3800, Australia; 7 School of Psychological Sciences, Monash University, Clayton 3800, Australia; 8 Francis Weld Peabody Society, Harvard Medical School, Boston, MA 02115, USA; 9 Minnesota Department of Health, St. Paul, MN 55164, USA; 10 Los Angeles County Department of Public Health, Los Angeles, CA 90007, USA; 11 Department of Medicine, University of Melbourne, Parkville 3010, Australia; 12 Division of Cancer Prevention and Control, Centers for Disease Control and Prevention, Atlanta, GA 30329, USA

**Keywords:** COVID-19, vaccination, adolescents, parents, school

## Abstract

*Objectives:* This study assessed the associations between parent intent to have their child receive the COVID-19 vaccination, and demographic factors and various child activities, including attendance at in-person education or childcare. *Methods:* Persons undergoing COVID-19 testing residing in Minnesota and Los Angeles County, California with children aged <12 years completed anonymous internet-based surveys between 10 May and 6 September 2021 to assess factors associated with intention to vaccinate their child. Factors influencing the parents’ decision to have their child attend in-person school or childcare were examined. Estimated adjusted odds rations (AORs, 95% CI) were computed between parents’ intentions regarding children’s COVID-19 vaccination and participation in school and extra-curricular activities using multinomial logistic regression. *Results:* Compared to parents intending to vaccinate their children (n = 4686 [77.2%]), those undecided (n = 874 [14.4%]) or without intention to vaccinate (n = 508 [8.4%]) tended to be younger, non-White, less educated, and themselves not vaccinated against COVID-19. Their children more commonly participated in sports (aOR:1.51 1.17–1.95) and in-person faith or community activities (aOR:4.71 3.62–6.11). A greater proportion of parents without intention to vaccinate (52.5%) indicated that they required no more information to make their decision in comparison to undecided parents (13.2%). They further indicated that additional information regarding vaccine safety and effectiveness would influence their decision. COVID-19 mitigation measures were the most common factors influencing parents’ decision to have their child attend in-person class or childcare. *Conclusions:* Several demographic and socioeconomic factors are associated with parents’ decision whether to vaccinate their <12-year-old children for COVID-19. Child participation in in-person activities was associated with parents’ intentions not to vaccinate. Tailored communications may be useful to inform parents’ decisions regarding the safety and effectiveness of vaccination.

## 1. Introduction

Child and adolescent COVID-19-related hospitalizations increased five-fold in the US between June and August 2021 and unvaccinated adolescents experienced elevated rates of hospitalization and serious illness compared to their vaccinated peers [1,2]. This increase occurred during the emergence of the SARS-CoV-2 Delta variant (B.1.617.2) and while schools and parents prepared for return to in-person school for the 2021–2022 school year after having transitioned to remote learning during the initial stages of the COVID-19 pandemic [3]. The Food and Drug Administration expanded the authorization of the Pfizer-BioNTech COVID-19 vaccine in May 2021 to include individuals aged 12–15 years. Although two doses of the vaccine are >90% effective against the Delta variant in preventing hospitalization, intensive care admission, or the use of life-support [4], only 46.4% of 16–17 year old and 37.0% of 12–15 year old adolescents had been fully vaccinated as of 1 September 2021 [5,6].

Given that child and adolescent vaccines are often administered at the discretion of their parents and guardians, child and adolescent vaccination rates differ by parental social and demographic characteristics. Prior to expanded vaccination authorization, adolescent (16–17 years old) COVID-19 vaccination rates were lower among those whose parents reported less than a bachelor’s degree or identified as female or having Hispanic ethnicity [7]. Parents determine whether their children are vaccinated against COVID-19. Therefore, a greater understanding of factors related to parental intent will inform opportunities to improve COVID-19 vaccination levels among children and adolescents, which would support a safer return to in-person activities.

The COVID-19 pandemic limited many activities that support child and adolescent development, such as attending school, sports, jobs, extra-curricular activities, social events, and religious services [8]. As highlighted in a recent review, this led to greater rates of depression and anxiety, which were partially mitigated by physical exercise and social connectiveness [9]. Accumulating evidence documents the transmission of SARS-CoV-2 during such activities and supports the need for a layered set of strategies, including vaccination to mitigate viral transmission and reduce the risk of severe illness [8,10,11,12,13]. Los Angeles County schools offer an example of an effective approach that supports child engagement in activities [12]. Schools in Los Angeles County were required to implement COVID-19 safety protocols (e.g., symptom screening, masking, physical distancing, cohorting, and contact tracing) for in-person learning during the 2020–2021 school year, which resulted in fewer school-associated COVID-19 cases when compared to community cases [12]. However, policies and requirements for prevention strategies vary by activity, jurisdiction, and level of COVID-19 transmission within a community [14]. Child and adolescent activities represent settings for potential interventions and opportunities to examine the potential increase in COVID-19 spread among younger population groups. To our knowledge, the relationship between parental vaccine intentions for their children and engagement in child and adolescent activities has not been documented. Therefore, the objective of this study was to assess the associations between parent intent to have their child or adolescent receive the COVID-19 vaccination series; demographic factors; and various child activities, including attendance at in-person education or childcare.

## 2. Methods

### 2.1. Data Collection

From 10 May–6 September 2021, adults aged ≥18 years who had recently tested for SARS-CoV-2 were sent text or e-mail messages from Los Angeles County (population: 10.04 million) Department of Health (LACDPH) or Minnesota (population: 5.6 million) Department of Health (MDH) with invitations to participate in a voluntary, anonymous, English-language internet-based survey as part of The COVID-19 Outbreak Public Evaluation (COPE) Initiative, in collaboration with public health officials from LACDPH, MDH, and the Centers for Disease Control and Prevention (CDC). Surveys were administered through Qualtrics, LLC (Provo, UT, and Seattle, WA, USA). Survey links were sent one time to persons tested for SARS-CoV-2 in the past 14 days at testing sites that reported to LACDH or MDH and for whom contact information was provided to the health departments. Information about the survey was available to recipients who opened the electronic survey link. This study was approved by the Monash University Human Research Ethics Committee.

Respondents provided demographic and socioeconomic information, including age, gender, race and ethnicity, education level, and household income level. To assess for the primary outcome variable of parental intent to vaccinate their children, parents or guardians of children aged <12 years were asked, “*When vaccination becomes available for children younger than 12 years old, do you plan to have your child get the COVID-19 vaccine?*” Responses were categorized as ‘’Intending to vaccinate’’, ‘’Undecided (“Maybe” or “Not sure”)’’ and ‘’Not intending to vaccinate’’.

Four other survey items were included in this analysis.

1. “What information would influence your decision to have your child get the COVID-19 vaccine?” Possible responses (more than one choice allowed) were:
  1. Information about whether the vaccine is safe for children;
  2. Evidence that vaccination prevents children from getting infected with the COVID-19 virus;
  3. Evidence that the vaccine prevents children from getting serious illness due to COVID-19;
  4. Evidence that vaccinated children are less likely to transmit the COVID-19 virus to others;
  5. I have all the information I need about COVID-19 vaccines.
2. “*Over the past month, did your child or children (select all that apply)”* Possible choices were:
  1. Attend in-person classes;
  2. Attend in-person childcare;
  3. Participate in a sports team;
  4. Participate in another in-person extracurricular activity;
  5. Have indoor visits with friends;
  6. Attend in-person religious or community activities;
  7. My child or children did not do any in-person activities.
3. For children attending school or childcare, parents were asked “*Which of the following helped to inform your decision to have your child or children attend in-person classes or childcare?*”
  Possible responses (more than one choice allowed) were:
  1. The school or childcare facility does not provide an option for virtual learning;
  2. I have challenges in providing virtual learning for my child or children;
  3. Low numbers of COVID-19 cases in my community;
  4. Vaccination of students, teachers, and staff;
  5. Availability of school-based COVID-19 testing;
  6. Symptom screening for students and staff;
  7. Indoor mask requirements for students and staff;
  8. Maintenance of other COVID-19 prevention measures, including physical distancing; use of physical barriers; and smaller classrooms.
4. For children NOT attending school or childcare, parents were asked “*Which of the following helped to inform your decision to have your child or children NOT attend in-person classes or childcare?*”
  Possible responses *(more than one choice allowed)* to this multiple-choice question were:
  1. My child or children would not be attending school or childcare anyway;
  2. The school or childcare facility does not provide an option for in-person learning;
  3. Low numbers of COVID-19 cases in my community;
  4. Vaccination of students, teachers, and staff before the school year begins;
  5. Availability of school-based COVID-19 testing;
  6. Symptom screening for students and staff;
  7. Indoor mask requirements for students and staff;
  8. Maintenance of other COVID-19 prevention measures, including physical distancing, use of physical barriers, and smaller classrooms;
  9. None of these.

### 2.2. Statistical Analysis

Our study team followed STROBE guidelines from the Equator Network Guidelines to report our results [15]. Demographic and social characteristics of the parent participants in the survey sample were described in total and stratified by parental intention to vaccinate their child for COVID-19. These characteristics included study area, age, gender, race and ethnicity, household income level, education level, employment status, parental COVID-19 vaccination status, median household members, and children in the household. Comparisons in percentages among parental intention to vaccinate categories were performed using χ^2^ square. We estimated the adjusted odds ratios (aOR) of the likelihood a child would participate in a certain activity based on parental intention to vaccinate their child using a stepwise multinomial logistic regression with forced entry of child activity. The reference group was “yes” to parental vaccination of their child(ren), and comparisons were made to “no” and “undecided”. Other factors and covariates considered for inclusion in the model were gender, race and ethnicity, age, study area, income, employment, and parental vaccination status. Parent education was omitted because of its close correlation with income (ϕ = 0.4). Point estimates are shown with 95% confidence intervals and *p*-values (α = 0.05). All analyses were conducted using SAS version 9.4 and IBM SPSS version 28. 

## 3. Results

**Figure 1:**
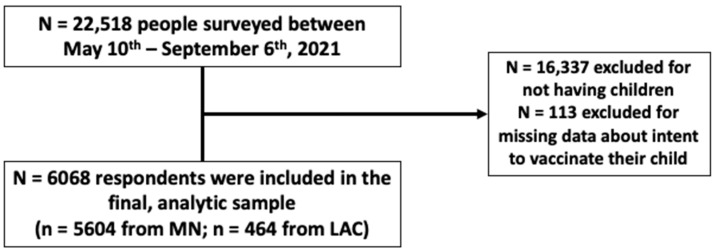
STROBE diagram.

Of 22,518 adults who completed online questionnaires between 10 May and 6 September 2021, 16,337 did not have children, and 113 who did have children did not report data about their intent to vaccinate their child; these respondents were excluded. After these exclusions, 6068 individuals were included in this analysis, including 5604 respondents in Minnesota and 464 in Los Angeles County, California (Figure 1). Among respondents, the median age was 40 years (range, 18 to 66 years), and 74% identified as female gender (Table 1). Combined race and ethnicity percentages differed between the two study areas. By residence, 87.7% of respondents in Minnesota identified as non-Hispanic White; 1.9% as non-Hispanic Black; and 3.5% as Hispanic or Latino ethnicity, any race or races. By comparison, in Los Angeles County, 31.0% identified as non-Hispanic White; 4.3% as non-Hispanic Black; and 48.7% as Hispanic or Latino, any race or races (*p*-value across groups < 0.001). More than half of respondents reported annual household income above $100,000, and 95% reported post-secondary education, with 42.6% possessing professional or post-graduate degrees. Most respondents (85.9%) were employed at the time of the survey. Respondents reported a median of two household members aged <12 years (minimum 1 to maximum 8).

Among 6068 parents or guardians of children aged <12 years, 4686 (77.2%) intended to have their children vaccinated against COVID-19 when available, 874 (14.4%) were undecided, and 508 (8.4%) did not intend to have their children vaccinated (Table 1). Residents of Los Angeles County were more likely to be undecided or not intending to vaccinate their children; vaccine hesitancy was higher among non-White parents compared to White, non-Hispanic in both states. Undecided parents and those with no intent to vaccinate tended to be younger, with higher percentages aged 18–29 years compared to parents intending to have their children vaccinated against COVID-19. Respondents reporting household incomes above $100,000 and possessing professional or post-graduate degrees more commonly reported intent to vaccinate their children. By self-reported COVID-19 vaccination status, 98.9% of respondents intending to vaccinate children had themselves received one or more COVID-19 vaccines, versus 84.0% of undecided parents and 36.6% of those not intending to have their children vaccinated.

Multivariate models revealed that demographic factors associated with no intention to vaccinate in comparison to intention to vaccinate were parent unvaccinated (aOR: 144.11, 95% CI: 102.95–201.71), decreasing age (aOR: 0.98, 95% CI: 0.96–0.99), and lower income level (aOR: 0.83, 95% CI: 0.74–0.95). Factors associated with indecision to vaccinate were parent unvaccinated (aOR: 13.62, 95% CI: 9.70–19.13), decreasing age (aOR: 0.97, 95% CI: 0.96–0.98), decreasing income (aOR: 0.76, 95% CI: 0.70–0.83), Los Angeles County residency (aOR: 1.43, 95% CI: 1.11–1.85), and having less than a college education (aOR: 1.51, 95% CI: 1.05–2.18).

As shown in Table 2, in comparison to parents who intended to vaccinate their children, parents with no intention to vaccinate had higher odds of reporting child participation in sports, other in-person extracurricular activities, indoor visits with friends, or in-person faith or community activities. Conversely, they had lower odds of reporting that their child engaged in no in-person activities. Undecided parents also had higher odds of reporting child indoor visits with friends and in-person faith or community activities but not of reporting participation in sports or other in-person extracurricular activities.

**Figure 2:**
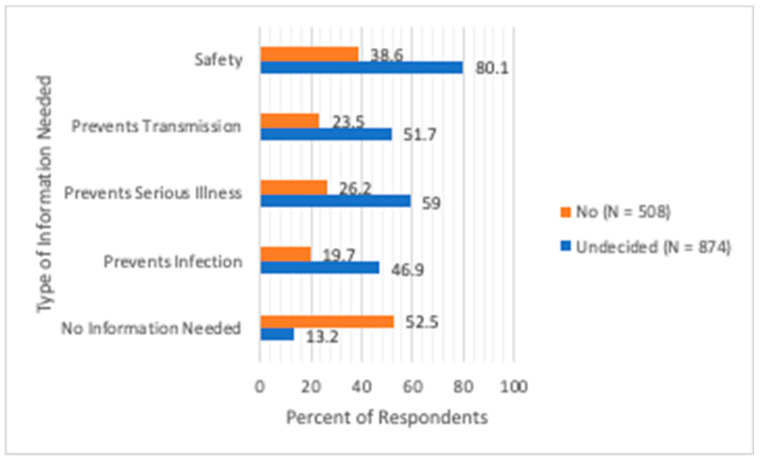
Percentages of undecided parents and parents not intending to vaccinate for whom additional information would influence their decision to have their child vaccinated for COVID-19.

When asked what information would influence their decision to vaccinate their children, 80.1% of undecided parents indicated that they sought more information about vaccine safety in children, 59% wanted evidence that vaccines would prevent children from serious COVID-19 illness, 51.7% wanted evidence that vaccination would prevent SARS-CoV-2 infections in children, and 46.9% wanted evidence for reduced SARS-CoV-2 transmission from vaccinated children. Overall, only 13.2% of undecided parents reported that they had all the information they needed about COVID-19 vaccines to make a decision about having their children vaccinated. In contrast, 38.6% of parents not intending to vaccinate their children wanted information about safety of vaccines in children, while 52.5% responded that they had all the information they needed about COVID-19 vaccines (Figure 2). Among those who indicated that they had all the information they needed, the proportion of Black, non-Hispanic parents was lower in comparison to other racial/ethnic groups (41.1% vs. 71.1%, *p* = 0.011). Otherwise, no other socio-demographic differences were found.

Overall, 31.1% and 35.4% of 6058 parent respondents reported their children had attended in-person classes or childcare, respectively, in the past month; percentages did not differ significantly by vaccination intentions (Table 2). As shown in Table 3, when parents were asked what factors influenced their decision to have children attend in-person classes or childcare, more than 50% identified indoor mask requirements for students and staff and vaccination of teachers or staff as important considerations with other factors mentioned less often. Additionally, most of these factors were more influential for parents who intended to vaccinate in comparison to those who were undecided or with no intention to vaccinate; percentages for undecided parents were more closely resembled responses of those with intention to vaccinate.

When asked what factors informed their decision to have their child not attend-in-person class or childcare, 41.0% of 2677 parents indicated that their child would not have attended school or childcare in any case, and 40.0% indicated that none of the factors listed would have influenced their choice. A small minority of parents indicated that the maintenance of COVID-19 prevention measures (13.9%); indoor masking requirements (11.7%); and vaccination status of teachers, staff, and students (9.4%) were considerations in their decision (Table 4). These factors were most influential for parents who were intending to vaccinate their children. Responses of parents who were undecided or with no intention to vaccinate were not different.

## 4. Discussion

This study examined factors associated with parental intent to vaccinate children and adolescents against COVID-19 among residents of Minnesota and Los Angeles County, California before or at the beginning of the 2021–2022 school year. Intentions to vaccinate children were associated with parents’ having themselves been vaccinated against COVID-19; older age; employment; higher education level; and higher household incomes. Parents of children who were engaged in extracurricular and religious activities had higher odds of reporting no intention or being hesitant to vaccinate their children aged <12 years. Most undecided parents reported that additional information regarding the safety and effectiveness of COVID-19 vaccination in children would influence their decision about vaccination of their children. Pandemic waves and potential for emergence of new SARS-CoV-2 variants are unpredictable. This study provides insights that suggest targeted efforts to inform subpopulations of vaccine-hesitant and resistant parents concerning the safety and effectiveness of COVID-19 vaccines for their children might improve uptake in this population. 

The factor most strongly associated with vaccination intention for children was parental vaccination status. Recent studies examining COVID-19 vaccination intention [16,17,18], including a recent international meta-analysis [19], also have found that parents vaccinated against COVID-19 are much more likely to have their children vaccinated as well. Studies indicate that parents who are vaccinated against COVID-19 are also more likely to be vaccinated against influenza [20] and give greater weight to physician recommendations [17].

Certain demographic and socioeconomic factors were associated with intention to vaccinate. Our finding that younger parents were more likely to be undecided or without intention to vaccinate is consistent with other studies as well [16,19,21]. Similarly, less education and lower income also were related to being undecided or without intention to vaccinate and are similar to previous investigations [18,19,21]. Together, these findings suggest that younger and more economically disadvantaged parents remain concerned and need additional safety and effectiveness information about vaccinating their children for COVID-19. We also observed that residents of Los Angeles County reported being more hesitant or less likely to vaccinate their children although this observation lost significance in multivariate analyses. Nevertheless, this still may reflect differences in attitudes toward COVID-19 vaccination or vaccination in general among these participants that were not captured by the demographic and socioeconomic factors surveyed.

Undecided parents and those not intending to vaccinate their children were more likely to report their child’s in-person attendance at faith and community activities, consistent with published reports of COVID-19 vaccine hesitancy among some religious groups [22,23,24]. The engagement of faith leaders (e.g., pastors, imams, youth group leaders, etc.) and outreach through churches and religious groups, as well as trusted scientific and medical authorities, may increase trust in vaccines and sources of information about the safety and effectiveness of the COVID-19 vaccines [25]. Ongoing efforts are needed to communicate the benefits of child vaccination through language tailored for specific groups.

Participation in school sports and indoor activities with other children have been associated with an increased risk of COVID-19 [26]. However, parents not intending to vaccinate their children were more likely to report children’s participation in these activities. The findings of this study demonstrating lower odds of intent to vaccinate children among parents who have children participating in such activities highlight the critical importance of educating parents about the benefits of vaccination for their children.

Undecided parents identified several areas in which tailored communication may influence decisions to vaccinate children, including more information on safety of COVID-19 and evidence of effectiveness against severe illness, infection, and potential for transmission from children to others. Information about the safety and effectiveness of COVID-19 vaccines approved for children may influence vaccine acceptance but may also require targeted delivery and the use of trusted sources of information [7,23,27,28]. One systematic review of communication and intervention strategies for addressing parental vaccine hesitancy towards childhood vaccines reported the most effective messengers to increase childhood vaccination uptake were health care professionals, family and friends, religious leaders, and the internet/social media; in contrast, health/government authorities were cited as barriers to trust among parents who were vaccine-hesitant [29]. Other successful strategies included having a “community parent vaccine advocate” to promote childhood vaccination, presenting vaccination as a default approach compared to an optional approach, and avoiding fear-based motivational anecdotes as some of the strongest techniques for decreasing hesitancy [29]. Despite interest in additional evidence of vaccine safety among both parents who did not intend to vaccinate and those who were hesitant to vaccinate their children, many nevertheless did not feel additional information about COVID-19 vaccines would influence their decision to vaccinate their children. Improving immunization coverage among children of these vaccine-hesitant parents may require outreach through different channels. 

Policy-related and education/mass communication efforts about the safety and effectiveness of COVID-19 vaccines for children could be modeled on successful interventions that were used for other childhood vaccinations. Legislative approaches have also increased child immunization rates [30,31,32]. Individual school districts could also add a COVID-19 vaccine mandate to the list of vaccines that are already required for most students to attend public school [33]. With political will and advocacy, more such policies could be enacted. 

Factors that parents reported as having influenced their decision to have their child attend in-person classes or childcare included indoor masking, teacher and staff vaccination status, and other COVID-19 mitigation measures. Support for indoor masking was also reported in a survey conducted of parents in Illinois, Michigan, and Ohio, with 61% favoring masks for school staff, but with less than 50% favoring masks for elementary school children [34]. Parents in this survey also supported increased COVID-19 mitigation measures. In contrast, we found that these factors were not frequently rated as important to parents who had decided not to have their children attend in-person classes or childcare. In a survey performed by the Brookings Institute, the most common reason (45% of parents) for their child not attending in-person schooling in April-May 2021 was that it was “Safer for child/family” [35]. Similar to our findings, COVID-19 mitigation strategies were selected by only a small number (e.g., “Adults at school not vaccinated”, 7%). Thus, while certain COVID-19 mitigation strategies favorably impact some parents’ decision to send their children to school, they have little influence on others. Further investigation is required to understand the reasoning of these latter parents inasmuch as virtual learning during the pandemic is associated with worse academic achievement [36] and worse social and mental health [37].

The results from our study suggest several strategies that should improve vaccination uptake. Considering that we found undecided parents as well as parents with no intention to vaccinate were more likely to have their children participate in sports and in extracurricular and faith-based activities, enlisting youth sports and activity organizations (e.g., Little League, youth soccer clubs, or boys and girls scouts) as well as local religious institutions in educational campaigns to highlight the benefits and safety of vaccination should lead to better child vaccination rates [38]. Furthermore, having on-site vaccination availability at youth sports events or activities or after religious services also may prove useful. Finally, given that vaccine hesitancy was higher in non-White parents, the use of promotores in Hispanic neighborhoods and community health advocates in other non-White neighborhoods to participate in vaccine educational campaigns should be a useful strategy [39].

These findings are subject to several limitations and should be interpreted in the context of the non-representative nature of a single survey administered before vaccines were recommended for children aged below 12 years. First, the survey did not include age of individual children, and thus associations between children’s ages, activities, and parent vaccination intentions could not be assessed. Second, the vaccination status of children was not collected—vaccination for older children and adolescents aged 12–15 years was approved in May 2021, and some children may have been vaccinated at the time of the survey. Third, actual decisions to vaccinate children when vaccines were recommended may differ from expressed intent to vaccinate; follow-up surveys to determine whether children were vaccinated have not been administered. Fourth, the survey collection included summer months when schools were closed. Parents completing surveys in July less commonly reported having children in classes during the past month. Fifth, the survey did not capture the time interval of SARS-CoV-2 Delta and Omicron variant waves of the pandemic, which may have changed intentions to vaccinate children. Sixth, survey respondents were more highly educated, had higher income, and more commonly reported having been vaccinated in Minnesota than in Los Angeles County; the latter had a higher proportion of racial/ethnic minorities. However, multivariate analyses did not find that the study area was associated with differences in intention to vaccinate. Seventh, our cohort was a non-representative voluntary sample of persons tested for COVID-19 and sent an invitation to participate in an anonymous survey by their health department. Thus, associations with intention to vaccinate can be compared with other survey respondents recruited through similar modalities but may not be generalizable to an unselected population of parents.

## 5. Conclusions

Parents with no intention to vaccinate their children against COVID-19 or who were undecided more commonly reported not having been vaccinated against COVID-19 themselves, younger age, and lesser education attainment. Child participation in in-person activities was associated with parents’ intentions not to vaccinate, and one of the strongest associations was with in-person faith or community activities. Responses from undecided parents indicate potential for tailored communication to inform parents’ decisions regarding safety and effectiveness of child vaccination. Furthermore, surveys of persons being tested for COVID-19 may provide useful information about current vaccination attitudes and contribute to the development of future vaccination promotion strategies. Layered public health interventions and policies are needed to improve COVID-19 immunization coverage among children and adolescents, which delays coverage in older age groups. Our findings should result in increased availability of trusted sources of information and advocates for COVID-19 vaccination of children, especially in non-White communities. This is particularly important because of low overall child vaccination rates (ages 5–11 years: 30.5%) [6] and evidence that death from COVID-19 may disproportionately impact non-White children and adolescents [40]. Furthermore, our results are informative for future public health communication strategies and vaccination promotion campaigns that are tailored for specific demographic and behavioral groups and will be applicable not only for COVID-19 vaccines but for future pathogens as well.

## Figures and Tables

**Table 1:** Demographic and social characteristics of parents by vaccination intent status.

Respondent Characteristics	Respondents with Children <12 Years (N = 6068)	Responses to Survey Question Regarding Intention to Vaccinate Children Aged <12 Years			
		Intending to Vaccinate Children (N = 4686)	Undecided to Vaccinate Children (N = 874)	Not Intending to Vaccinate Children (N = 508)	*p*-Value
**Study area—n (%)**					<0.001
Los Angeles County, California	464 (7.6)	300 (64.7)	108 (23.3)	56 (12.1)	
State of Minnesota	5604 (92.4)	4386 (78.3)	766 (13.7)	452 (8.1)	
Combined MN and LA	6068 (100)	4686 (77.2)	874 (14.4)	508 (8.4)	
**Age in years, median—years (min, max)**	40 (18, 66)	40 (18, 65)	37 (18, 65)	37 (18, 66)	<0.001
**Age group in years—n (%)**					<0.01
18–29	399 (6.6)	128 (4.6)	62 (11.8)	53 (17.3)	
30–44	4192 (69.1)	1842 (65.6)	369 (70.0)	192 (62.5)	
45–59	1392 (22.9)	804 (28.6)	82 (15.6)	58 (18.9)	
≥60	85 (1.4)	36 (1.3)	14 (2.7)	4 (1.3)	
**Gender—n (%)**					0.007
Male	1605 (26.5)	1283 (27.4)	217 (24.8)	105 (20.7)	
Female	4420 (72.8)	3367 (71.9)	654 (74.8)	399 (78.5)	
Transgender/Other	43 (0.7)	36 (0.8)	3 (0.3)	4 (0.8)	
**Combined Race and Ethnicity—n (%)**					<0.001
Asian, non-Hispanic	228 (3.8)	179 (3.8)	40 (4.6)	9 (1.8)	
Black, non-Hispanic	125 (2.1)	70 (1.5)	36 (4.1)	19 (3.7)	
Hispanic or Latino, any race or races	423 (7.0)	274 (5.8)	92 (10.5)	57 (10.5)	
Multiple races, non-Hispanic	147 (2.4)	109 (2.3)	23 (2.6)	15 (3.0)	
Other race, non-Hispanic	84 (1.4)	49 (1.0)	13 (1.5)	22 (4.3)	
White, non-Hispanic	5061 (83.4)	4005 (79.1)	670 (76.7)	386 (76.0)	
**Household Income—n (%)**					<0.001
Less than $25,000	215 (3.5)	105 (2.2)	71 (8.1)	39 (7.7)	
$25,000–$49,000	505 (8.3)	297 (6.3)	124 (14.2)	84 (16.5)	
$50,000–$99,999	1445 (23.8)	1046 (22.3)	236 (27.0)	163 (32.1)	
$100,000 or more	3576 (58.9)	3024 (64.5)	375 (42.9)	177 (34.8)	
Unknown	327 (5.4)	214 (4.6)	68 (7.8)	45 (8.9)	
**Education—n (%)**					<0.01
High school degree or less	221 (3.6)	106 (2.3)	70 (8.0)	45 (8.9)	
Some college or bachelor’s degree	3261 (53.7)	2309 (49.3)	581 (66.5)	371 (73.0)	
Professional or doctoral degree	2586 (42.6)	2271 (48.5)	223 (25.5)	92 (18.1)	
**Currently employed—n (%)**					0.004
Yes	5213 (85.9)	4062 (86.7)	733 (83.9)	418 (82.3)	
No	855 (14.1)	624 (13.3)	141 (16.1)	90 (17.7)	
**Received one or more COVID-19 vaccines—n (%)**					<0.001
Yes	5555 (91.5)	4635 (98.9)	734 (84.0)	186 (36.6)	
No	513 (8.5)	51 (1.1)	140 (16.0)	322 (63.4)	
**Number of household members—mean (min, max)**	4 (2, 16)	4 (2, 16)	4 (2, 16)	503 (2, 14)	<0.001
**Number of children aged <12 years—median (min, max)**	2 (1, 8)	2 (1, 8)	2 (1, 8)	2 (1, 7)	<0.01

**Table 2:** Associations between parental intention to vaccinate their children before the 2021–2022 school year and child activities, May–September 2021.

Child Activities	Intention to Vaccinate Children (N = 4686) n (%)	Undecided to Vaccinate Children (N = 874) n (%)	aOR (95% CI) *	No Intention to Vaccinate Children (N = 508) n (%)	aOR (95% CI) *
Attended in-person classes	1453 (31.0)	266 (30.4)	1.04 (0.88, 1.23)	163 (32.1)	1.16 (0.90, 1.50)
Attended in-person childcare	1669 (35.6)	319 (36.5)	1.17 (0.99, 1.38)	154 (30.3)	1.03 (0.80, 1.34)
Participated in sports	1819 (38.8)	277 (31.7)	1.01 (0.86, 1.19)	190 (37.4)	**1.51 (1.17, 1.95)**
Other in-person extracurricular activities	1932 (31.8)	286 (32.7)	0.92 (0.79, 1.09)	195 (38.4)	**1.35 (1.05, 1.73)**
Indoor visits with friends	2629 (56.1)	471 (53.9)	**1.19 (1.02, 1.39)**	336 (66.1)	**2.31 (1.78, 2.99)**
In-person faith or community activities	670 (14.3)	194 (22.2)	**1.90 (1.58, 2.29)**	206 (40.6)	**4.71 (3.62, 6.11)**
No in-person activities	464 (9.9)	126 (14.4)	0.92 (0.72, 1.17)	56 (11.0)	**0.55 (0.37, 0.81)**

* Adjusted odds ratios (aORs) were estimated and adjusted for gender, race and ethnicity, age, income, study area, employment, and parental vaccination status. All point estimates are shown with 95% confidence intervals; *p* ≤ 0.05 indicated in boldface.

**Table 3:** Percentages of parents endorsing factors that informed a decision to have their children attend in-person class or childcare stratified by intention to vaccinate.

	Intending to Vaccinate (N = 2686)	Undecided (N = 490)	No Intention to Vaccinate (N = 265)	Overall (N = 3441)
No Virtual Learning Option	16.7	16.9	15.1	16.6
Parental Challenges to Provide Virtual Learning	19.2	16.9	11.7 ^a,c^	18.3
Low Local COVID Levels	28.5	22.2 ^a^	18.1 ^b^	26.8
Vaccinated Teachers/Staff/Students	63.6	43.5 ^b^	13.6 ^b,d^	56.9
Available Onsite COVID Testing	8.2	5.9	3.4 ^a^	7.5
Onsite Symptoms Screening	40.9	30.0 ^b^	17.7 ^b,d^	37.6
Indoor Masking Requirements for Staff/Students	59.6	36.1 ^b^	19.6 ^b,d^	53.2
Other Prevention Measures *	50.0	34.7 ^b^	18.9 ^b,d^	45.4
None of the above	23.3	46.4 ^b^	9.6 ^b,d^	14.4

^a^*p* ≤ 0.05; ^b^ *p* ≤ 0.01 vs. intending to vaccinate. ^c^ *p* = 0.055; ^d^ *p* ≤ 0.01 undecided vs. no intention to vaccinate. * e.g., physical distancing, use of physical barriers and smaller classrooms.

**Table 4:** Percentages of parents endorsing factors that informed a decision to have their children not attend in-person class or childcare stratified by intention to vaccinate **.

	Intending to Vaccinate (N = 2000)	Undecided (N = 384)	No Intention to Vaccinate ^d^ (N = 243)	Overall (N = 2627)
Indoor Masking Requirements for Staff/Students	13.3	6.5 ^c^	6.6 ^c^	11.7
Onsite Symptoms Screening	7.7	3.9 ^c^	3.3 ^c^	6.7
Available Onsite COVID Testing	4.8	3.1 ^a^	1.6 ^b^	4.3
Vaccinated Teachers/Staff/Students	11.1	4.7 ^c^	2.5 ^c^	9.4
Low Local COVID Levels	4.8	3.9	2.1	4.4
No In-Person Learning	2.5	3.1	2.1	2.6
Additional Prevention Measures *	15.5	8.9 ^c^	8.6 ^c^	13.9
Factors Other Than Above	36.2	52.1 ^c^	52.7 ^c^	40.0
Would Not attend School/Childcare Anyway	43.5	32.8 ^c^	34.2 ^b^	41.0

^a^*p* = 0.087; ^b^ *p* ≤ 0.01; and ^c^ *p* < 0.001 vs. intending to vaccinate. ^d^ No factors were significantly different between undecided and no intention to vaccinate. * e.g., physical distancing, use of physical barriers and smaller classrooms. ** Data collection (May–September, 2021) included summer months when schools were closed for summer break (June–July 2021).

## Data Availability

Data are available from the corresponding author on reasonable request.
